# Should We Pre-date the Beginning of Scientific Psychology to 1787?

**DOI:** 10.3389/fpsyg.2018.02481

**Published:** 2018-12-06

**Authors:** Roland Pfister, Katharina A. Schwarz

**Affiliations:** Department of Psychology, University of Würzburg, Würzburg, Germany

**Keywords:** history of psychology, scientific psychology, 18th century, Ueberwasser, Wundt

## Psychology in the Eighteenth Century

The late eighteenth century was a remarkable period for psychology (Vidal, 2000, 2011; Schwarz and Pfister, 2016). This was especially true for the Prince-Bishopric of Münster, Germany, where a new era dawned with an extensive political, economic, and educational reform (Esser, 1842; Brühl, 1905) which would ultimately lead to the establishment of psychology as an independent discipline at the newly founded University of Münster. But were these developments groundbreaking enough to advocate for pre-dating the beginnings of scientific psychology?^1^

In the years 1762–1780, the newly appointed minister Franz Friedrich von Fürstenberg set out to modernize the state's agriculture, public budget, police, military, jurisdiction, and education (Esser, 1842). The educational part of these reforms included a seminal commitment to psychology, with Fürstenberg declaring psychology as a “core science” to be taught at every school within the territory (von Fürstenberg, 1776). Soon after, Fürstenberg was granted the right to establish a university in the city of Münster (Pieper, 1902). Following traditional procedures, the foundation of the university was authorized by Pope Clement XIV., and later ratified by Holy Roman Emperor Joseph II. The university itself was inaugurated in 1780 and comprised of four canonical faculties: Philosophy, providing general education for all students, and the three applied faculties of Jurisdiction, Medicine, and Theology.

The first professor to teach philosophy in Münster, Aloys Havichorst (1737–1783), implemented lessons on psychology as part of his classes on metaphysics, thus following common scientific taxonomies of that time (Meier, 1757). This state of affairs changed, however, when his successor Ferdinand Ueberwasser (1752–1812) was appointed professor of philosophy in 1783 (Schwarz and Pfister, 2016; cf. also Carus, 1808; Bödeker, 2003).

In contrast to typical views of his contemporaries, Ueberwasser did not subsume psychology under the field of metaphysics, but rather followed Fürstenberg's plea for conceptualizing psychology as a science of its own. Accordingly, he changed the denomination of his professorship into *Professor of Empirical Psychology and Logic* (Ger.: “Professor für empirische Psychologie und Logik;” later also: Psychology and Metaphysics; Schwarz and Pfister, 2016). Finally, in 1787 he published a remarkable textbook entitled “*Instructions for the regular study of empirical psychology for candidates of philosophy at the University of Münster*” (Ger.: “Anweisungen zum regelmäßigen Studium der Empirischen Psychologie für die Candidaten der Philosophie zu Münster”).

In his textbook, Ueberwasser outlined the methodological foundations of scientific psychology, followed by a broad overview of relevant psychological phenomena ranging from perception and memory to motivation, emotion, and volition (Ueberwasser, 1787; see also Schwarz and Pfister, 2016, for details on Ueberwasser's psychology). At 1787, scientific psychology thus seems to have made a first appearance as an independent discipline: It was officially represented by a state-funded professorship, the university had integrated psychological courses in its curriculum, and an early manifesto outlined structure and scope of the discipline.

## Pre-Dating Psychology?

Ueberwasser's and Fürstenberg's commitment to psychology as an independent scientific discipline clearly exceeds the emphasis that is typically found in academic discussions of empirical psychological during the late eighteenth and early nineteenth century (Meier, 1757; Schmid, 1791; Herbart, 1824/1825). Furthermore, these developments seem to parallel the well-known, later achievements of Wilhelm Wundt (1832-1920) with his *Lectures on Human and Animal Psychology* (Wundt, 1863; Ger.: “*Vorlesungen über die Menschen- und Thierseele*”) and his official foundation of the Leipzig laboratory in 1879 (Wontorra et al., 2004). That is, while other pioneers of a scientific and experimental approach to psychological questions such as Weber (1795–1878), Fechner (1801–1887) or Helmholtz (1821–1894) did not consider themselves psychologists, Ueberwasser and Wundt both attempted to establish psychology as an independent field of study, explicitly portraying themselves as psychologists.

In addition to these structural similarities, Ueberwasser's and Wundt's conceptions of scientific psychology also converge on a number of critical theoretical aspects. For instance, both emphasize the utility of physiological processes for understanding psychology, while simultaneously arguing against physiological reductionism. Thus, physiology is mainly seen as providing methods and approaches for testing and validating psychological accounts. Both also favor clear connections between philosophy and psychology, thus arguing against a pure natural science approach to psychological phenomena but rather advocating a unique approach to psychological questions. Finally, both promote repeated observation in controlled, structured contexts to establish generalizable and replicable findings.^2^

These commonalities beg the question of whether Ueberwasser's legacy should be construed either as an early precursor of contemporary psychology or, alternatively, whether it deserves even stronger recognition in terms of a founding date of scientific psychology?

We believe that—despite being outstanding and unparalleled at their time—Ueberwasser's achievements still fall short of a true foundation of scientific psychology, for the sole reason that his works did not establish a continued tradition of scientific psychology in the academic system nor do they seem to have been pivotal at inspiring later developments, particularly early psychophysical work (e.g., Fechner, 1860), or Wundt's comprehensive approach to psychology (cf. Luna, 2016). Rather, Ueberwasser's legacy seems to have disappeared relatively quickly, so that only few references to his work appear even in writings that were published shortly after his death, i.e., at the outset of the nineteenth century (Carus, 1808; Biunde, 1832).

What were the reasons for these developments? Prior to his academic appointments, Ueberwasser had been a novice in the Society of Jesus, thus pursuing a clerical career. After the society had been banned by papal decree in 1773, ex-Jesuits often developed close ties to selected members of the local catholic elites. This was also true for Ueberwasser, as Fürstenberg was a decisively catholic statesman and an active promoter of former Jesuits; he thus ensured that several former Jesuits attained positions in renowned schools, the local military academy, or the University of Münster. Moreover, Ueberwasser was long-standing member of the “Circle of Münster,” a small, close-knit society around Fürstenberg and Princess Amalia Gallitzin (Bödeker, 2003). The Circle of Münster existed from 1779/1780 to 1806 and included a number of eminent former Jesuits. Thus, the circle's members and their immediate academic fellows, especially Ueberwasser's designated successor, Georg Laymann, were dedicatedly catholic and their agenda clearly followed the spirit of the Catholic Enlightenment (Niehaus, 1998).

Even though this arrangement had paved the way for the early emancipation of psychology in Münster it was also pivotal for its demise. At the beginning of the nineteenth century, the Revolutionary Wars (1792–1802) and the later Napoleonic Wars (1803–1815) dominated European politics and resulted in Münster falling to Prussia, i.e., a declared protestant power (Pieper, 1902; Elstrodt and Schmitz, 2013). In the following years, the achievements of Fürsternberg's educational reforms were overturned and the University of Münster was closed already in 1818. These developments did not allow for Ueberwasser's legacy to strive, and his former pupils arranged with the new order by moving to clerical schools in the Münster area (Hegel, 1971).

It seems well possible that—without being overshadowed by such drastic political events—Ueberwasser's legacy might have outlasted his death in 1812. To our knowledge this was not the case however (Biunde, 1832) so that it would take another 100 years after the publication of Ueberwasser's “*Instructions for the regular study of empirical psychology*” for psychology to finally establish a foothold in the system of the sciences. Until there is evidence to link Ueberwassers achievements to any of the developments that paved the way for modern scientific, experimental psychology, we therefore argue that the founding moment of modern scientific psychology should still be dated to the foundation of Wundt's laboratory in 1879.^3^ This argument notwithstanding, Ueberwasser still seems to be the first academic to have considered himself a psychologist so that Ueberwasser as well as Fürstenberg certainly deserve prominent places in the (pre-) history of scientific psychology.

## Author Contributions

Both authors were involved in the historiographical research leading to this opinion paper. RP drafted the first version of the manuscript and KS provided critical revisions.

### Conflict of Interest Statement

The authors declare that the research was conducted in the absence of any commercial or financial relationships that could be construed as a potential conflict of interest.

## Footnotes

^1^The term “scientific psychology” is intended to refer to the Western understanding of experimental psychology for which Wundt's establishing of a psychological laboratory is typically portrayed as the discipline's founding moment. We use the term “scientific psychology” rather than “experimental psychology” to follow Ueberwasser's terminology.

^2^Note that Ueberwasser, in contrast to Wundt, likely did not carry out any studies that would qualify as experiments in the current sense of the term. Rather, he urged his students to perform repeated self-observation in different contexts and to catalogue their observations in structured tables (Schwarz and Pfister, 2016).

^3^On a more general note, one could ask whether scientific psychology in the Western spirit should take a premier spot relative to other (mostly non-quantitative and non-experimental) approaches to psychological questions which have emerged in different non-Western cultures. We believe that this question is viable, although it exceeds the scope of the present argument. We are grateful to the reviewer for raising this point.
